# Novel drug therapy of acute hepatic failure induced in rats by a combination of tadalafil and Lepidium sativum

**DOI:** 10.1186/s12906-024-04406-4

**Published:** 2024-02-27

**Authors:** Mahmoud S. Sabra, Ahmed A. Mohammed, Khaled M. Ahmed Hassanein, Ahmed A. N. Ahmed, Dalia Hassan, Ebtsam S. Abdel-lah

**Affiliations:** 1 Pharmacology Department, Faculty of Veterinary Medicine, Assiut University, Assiut, 71526 Egypt; 2 Department of animal and poultry behavior and management, Faculty of Veterinary Medicine, Assiut University, Assiut, 71526 Egypt; 3 Pathology and Clinical Pathology Department, Faculty of Veterinary Medicine, Assiut University, Assiut, 71526 Egypt; 4 Pharmacology Department, Faculty of Medicine, Al-Azhar University, Assiut branch, Assiut, 71526 Egypt; 5 Department of animal and poultry hygiene and environmental sanitation, Faculty of Veterinary Medicine, Assiut University, Assiut, 71526 Egypt

**Keywords:** Tadalafil, LS, CCL_4_, Acute liver injury, Oxidative stress, Inflammatory mediators, NF-κB

## Abstract

**Background:**

Hepatocyte death and a systemic inflammatory response are the outcome of a complex chain of events mediated by numerous inflammatory cells and chemical mediators. The point of this study was to find out if tadalafil and/or Lepidium sativum (*L. sativum*) could help people who have been exposed to carbon tetrachloride (CCL_4_) and are experiencing acute moderate liver failure. This was especially true when the two were used together.

**Method and materials:**

To cause mild liver failure 24 h before sacrifice, a single oral dosage of CCL_4_ (2.5 mL/kg b.w.) (50% in olive oil) was utilized. Furthermore, immunohistochemical expression of nuclear factor kappa B (NF-κB) as well as histological abnormalities were performed on liver tissue.

**Results:**

The results showed that tadalafil and/or *L. sativum*, especially in combination, performed well to cure acute mild liver failure caused by CCL_4_. This was demonstrated by a decrease in NF-κB expression in the liver tissue and an improvement in organ damage markers observed in the blood and liver tissues. Furthermore, such therapy reduced interleukin1 beta (IL-1β) and tumor necrosis factor-alpha (TNF-α) levels in the liver tissue. It’s worth noting that the tested combination resulted in greater liver improvement.

**Conclusions:**

According to the findings, tadalafil and *L. sativum*, particularly in combination, have the ability to protect the liver from the negative effects of CCL_4_ exposure. Because of its capacity to improve liver function, restore redox equilibrium, and decrease inflammatory mediators, it is a prospective option for mitigating the negative effects of common environmental pollutants such as CCL_4_.

## Background

A complicated chain of events mediated by various inflammatory cells and molecular mediators results in hepatocyte death and a systemic inflammatory response. The duration of the ischemia and the underlying liver disease determine the severity of the inflammatory reaction and organ dysfunction [1]. Many pharmacological treatments have been discovered to protect the liver from damage. These agents include antioxidants, ozone, adenosine agonists, nitric oxide (NO) donors, sildenafil, and vardenafil [2]. CCl_4_ is a xenobiotic industrial solvent used to cause chemical hepatitis and liver damage in animals. Carbon tetrachloride-induced liver damage is the most commonly used experimental model for assessing a drug’s hepatoprotective efficacy. A single dose of CCl_4_, a potent hepatotoxic xenobiotic, causes acute liver necrosis and steatosis [3, 4].

Mechanistic studies showed that cytochrome P2E1 is a key part of the proposed mode of action because it changes CCl_4_ into highly reactive free radical metabolites. Trichloromethyl and trichloromethyl peroxy are very reactive free radicals that can bind to biological macromolecules and help membrane phospholipid fatty acids form covalent bonds. By damaging polyunsaturated fatty acids within cell membranes, free radicals cause lipid peroxidation, resulting in a chain reaction of free radicals [4]. When the liver fails because of CCL_4_, an imbalance between reactive oxygen species and antioxidant defense causes cells to malfunction and liver necrosis [5]. CCl_4_ treatment greatly enhances hepatic enzyme release, cytochrome P450 degradation, lipid peroxidation products, and an inflammatory response [6]. Its method of action is seen in the liver cytochrome P450 system’s reductive dehalogenation, which produces trichloromethyl free radicals, which quickly combine with molecular oxygen to form trichloromethyl peroxy radicals [7].

Carbon tetrachloride is a prevalent contaminant in the environment. Workers are particularly vulnerable to high-level exposure through inhalation and skin contact [8]. On the other hand, the general population may be exposed to low quantities of CCL_4_ by atmospheric inhalation [9]. Among the several methods of administration of CCl_4_, the orogastric route is the most commonly employed since it offers significant benefits over other options. Oral administration of CCl_4_ in particular needs minimal amounts of CCl_4_ and enables direct transport to the liver via the portal vein, reducing extrahepatic effects due to the selective buildup of CCl_4_ in the liver [10].

According to animal welfare science, there is a strong link between animal welfare and animal health conditions [11]. Since animal welfare discipline is mostly dependent on behavior. As a result, employing animal models to research the therapeutic targets of innovative pharmaceuticals and herbal treatments must be supported by a thorough examination of normal animal behavior using various behavioral assays. This is an important technique in liver illnesses since it provides us with a full description of the many phases of liver problems and therapy, as well as how far they can impact animal behavior [12].

Tadalafil is a potent and selective phosphodiesterase type-5 (PDE5) inhibitor that was initially studied as a potential antianginal drug but has since grown in popularity in the treatment of erectile dysfunction and pulmonary arterial hypertension. Phosphodiesterases inactivate cyclic guanosine monophosphate (cGMP) to GMP. PDE inhibition enhances and prolongs the cellular responses to NO and its derivatives that cause vasodilation [13]. It also has clinically approved immunomodulatory action [14]. It was also utilized as a medical therapy for portal hypertension in patients with compensated cirrhosis, and it may have long-term benefits in compensated cirrhosis cases [15], cardioprotection in mice [16], pulmonary hypertension treatment [17], chronic renal failure prevention [18], treatment of Alzheimer’s disease, lower urinary tract symptoms caused by nonmalignant prostate hyperplasia, and irritable bowel syndrome [19–21].

Mansour, Salama [22] study sought to investigate the protective effect of tadalafil, a PDE5 inhibitor, against thioacetamide-induced liver fibrosis. In their study, tadalafil pretreatment protected against thioacetamide-induced liver fibrosis in a dose-dependent manner, as evidenced by the reduction of inflammatory and fibrotic indices. A Bektas, Karakaya [23] study also found that high doses of tadalafil (10 mg/kg) and pentoxifylline (40 mg/kg) have the best protective effect against ischemia reperfusion-induced liver tissue damage. Furthermore, PDE5 inhibitors have been shown to have a potentially promising role in the treatment of inflammatory processes [24] as well as anti-fibrotic effects [25]. Activation of cGMP-dependent protein kinases causes vasodilation, anti-inflammatory, and anti-proliferative effects, as well as a decrease in collagen synthesis [26–28].

Natural medicinal products may hold the key to natural xenobiotic/drug hepatoprotection [29]. *L. sativum* is an edible annual herb that grows wild in the Brassicaceae family. *L. sativum* is a medicinal plant that originated in Egypt and the Middle East and is now grown all over the world. *L. sativum* is used in traditional medicine to treat inflammatory disorders such as diabetes, arthritis, traumatic injuries, and hepatitis [30].

Various in vitro biological effects of *L. sativum* extract have been reported, including antioxidant, anti-inflammatory, antidiarrheal, antimicrobial, antispasmodic, and hepatoprotective action against oxidative damage, and thus have a high potential for use as herbal hepatoprotective or dietary supplements [30]. Literature on phytochemical studies of *L. sativum* revealed the presence of benzyl isothiocyanate, flavonoids, tannins, triterpenes, alkaloids, sterols, and glucosinolates, all of which have antioxidant, anti-inflammatory, analgesic, and hepatoprotective properties [31–33].

According to a study conducted by Al-Asmari, Athar [34], *L. sativum* seeds have enhanced hepatoprotective activity against CCL_4_ (1 mL/kg b.w. via the intraperitoneal route)-induced liver failure in rats, which could be attributed to their antioxidant activity combined with the presence of anti-inflammatory compounds in *L. sativum* extract. Furthermore, Rajab and Ali [35] study found that *L. sativum* could be used to prevent hepatotoxicity caused by CCL_4_ (1 mL/kg b.w. via the intraperitoneal route 2 times weekly for 12 weeks) in rats through anti-oxidant and anti-inflammatory effects. As a result, the purpose of this work is to discover more about the therapeutic potential of tadalafil, *L. sativum*, and especially their combinations against new orogastric CCl_4_-induced moderate liver damage in rats using biochemical, histological, and immunohistochemical techniques.

## Materials and methods

### Collection of plant material and extract preparation

The *L. sativum* used in this study was obtained from a local market in Assiut, Egypt, and authenticated by a medicinal plant expert at the Processing and Extraction Unit of Medicinal Plants, Faculty of Agriculture, Assiut University, Egypt. The seeds of *L. sativum* were harvested throughout the winter season. The extraction was carried out using the Soxhlet apparatus. Sixty grams of dry seeds were ground into a coarse powder. Then, 60 g of finely crushed seeds were mixed with 600 mL of ethanol. The extraction was carried out for 6–8 h, or until all of the soluble constituents were dissolved in the solvent. To obtain semisolid masses, the extract was filtered and evaporated in a rotary evaporator (Buchi, Switzerland; temperature: 60 °C; pressure: 175 mbar). The resulting extract was collected and stored at 4 °C until further use [34]. The overall yield was 14.1% w/w.

### Animal model and induction of acute liver failure

Following approval from the Ethics Committee (approval no: 06/2023/0042), this study was carried out in accordance with the guide for the care and handling of laboratory animals and in accordance with ARRIVE guidelines. This study used adult male albino rats aged 10–12 weeks. They were obtained from Egypt’s animal house’s faculty of veterinary medicine in Assiut. They weighed approximately 250 g. They were kept in a clean room with lights on from 5.00 AM to 7.00 PM and temperatures ranging from 27 to 32 °C. Commercial pelleted feed contains (protein 21%, fat 3%, fibre 5%, ash 8%, calcium 0.8%, phosphorous 0.4%, and silica 1.3% w/w) and water *ad libitum* were given to the animals.

A single oral administration of carbon tetra chloride (2.5 mL/kg b.w.) (50% in olive oil) was used to induce moderate liver failure 24 h before sacrifice [36].

### Behavioral assessment

The raised plus maze test was used to study anxiety-related behaviors in rats in order to determine the potential influence of each treatment on animal patterns [37]. Three duplicates of each treatment were used to record their behavioral patterns on the same day of each medication injection. The wooden gadget had open and closed arms, with open arms without borders and closed arms with 50 cm-high edges [38]. Rats were placed in the middle of the device for 5 min, and time spent in each arm was recorded. After each test, 70% alcohol was used to clean and disinfect the equipment.

### Experimental groups

They were divided into five groups (*n* = 6 each) at random. Group I served as the control rats; they were given 1 mL/kg body weight (b.w.) of olive oil intraperitoneally and the normal saline by oral gavage for 7 days. Group II was given 2.5 mL/kg b.w. of CCl_4_ (50% in olive oil) orally 24 h before sacrifice. *L. sativum* extract 300 mg/kg b.w [39]. was administered orally to Group III for one week. For one week, Group IV was given a moderate dose of tadalafil (5 mg/kg b.w.) [23] orally. For one week, Group V was given a combination of tadalafil and *L. sativum* extract. Except for the control group, all animals were administered CCl_4_ orally immediately before treatment, 24 h before sacrifice.

### Blood collection, sample preparation and storage

After an overnight fast (approximately 12-14 h), blood samples were taken from the retro-orbital venous plexus of anaesthetized rats via the eye canthus. Before scarification, rats were euthanized as follows: rats were fully sedated by breathing 5% isoflurane. Blood samples were then taken from the eye-canthus. When rats did not respond to head and limb stimulation, they were rapidly killed by cervical dislocation. If rats ceased breathing and did not respond to systemic stimulation after 10 s of cervical dislocation, they were ruled dead [40]. Blood samples were collected in plain tubes, centrifuged for 15 min at 4000 rpm to obtain serum, and stored at -20 °C until analysis. The lungs were quickly dissected, removed, and cleaned with a 0.9% NaCl solution. Liver tissues were minced and homogenized (10% w/v) in an ice-cold potassium phosphate buffer (0.1 M, pH 7.4). The homogenate was centrifuged at 3000 g for 10 min at 4 degrees Celsius, and the resulting supernatant was used to measure oxidative stress, antioxidants, and inflammatory markers. Other parts of the liver tissue were examined histopathologically and immunohistochemically.

### Determination of liver function markers

#### Determination of serum aspartate aminotransferase (AST) activity

The AST enzyme activity assay kit was used to determine AST activity, a marker for liver function. The enzyme, a PLP-dependent enzyme, converts aspartate and keto-glutarate to oxaloacetate and glutamate aspartate, producing a colorimetric active product proportional to its enzymatic activity. The kinetic method was described by Reitman and Frankel [41].

#### Determination of serum alanine aminotransferase (ALT) activity

Alanine aminotransferase (ALT) activity was determined using a kinetic approach developed by Reitman and Frankel [41]. GPT, a pyridoxal phosphate-dependent enzyme, catalyzes the reversible transfer of amino groups from alanine to keto-glutarate, producing pyruvate and glutamate and a colorimetric active product proportionate to the pyruvate produced.

### Determination of serum total protein activity

Gornall, Bardawill [42] established a colorimetric technique for determining serum proteins. According to the reagent kit, the protein creates a violet hue in the presence of alkaline cupric sulphate, the intensity of which is proportional to their concentration.

### Determination of serum albumin level

The content of serum albumin was evaluated using the colorimetric technique published by [43]. The assay was carried out in accordance with the reagent kits, which rely on the formation of an albumin/bromcresol-green complex at pH 3.8 and photometric absorbance measurement.

### Determination of serum alkaline phosphates (ALP) activity

The colorimetric approach published by [44] was used to detect serum ALP activity. The experiment was carried out using reagent kits that rely on the conversion of phenyl phosphate into phenol in the presence of alkaline phosphatase, and the freed phenol is quantified calorimetrically in the presence of 4-aminophenazone and potassium ferricyanide.

### Determination of serum gamma-glutamyl transferase (GGT) level

The kinetic technique established by Bergmeyer, Herder [45] was used to measure serum GGT activity. The test was carried out in accordance with the reagent kits. The gamma-glutamyl group is transferred from the donor substrate (L-gamma-glutamyl-3-carboxy-4-nitroanilide) to the glycylglycine acceptor via GGT, yielding 3-carboxy-4-nitroaniline. The absorbance rate is directly proportional to the GGT in the sample.

### Determination of serum bilirubin level

The colorimetric approach reported by Belfield and Goldberg [37] was used to detect serum bilirubin. The test was carried out in accordance with the reagent kits, which are based on the reaction between bilirubin and the diazonium salt of sulphanilic acid, which produces azobiliruin, which has a maximum absorption at 535 nm in an acid medium. Total bilirubin participates in the reaction in the presence of dimethylsulfoxide (DMSO), whereas only conjugated bilirubin responds in the absence of DMSO.

### Measurement of liver tissue lipid peroxidation

The malondialdehyde (MDA) level in the liver tissue homogenate was determined using the technique described by [46]. After a colorimetric reaction with thiobarbituric acid, the MDA level was determined spectrophotometrically. MDA was measured since it is a useful marker for oxidative stress and lipid peroxidation.

### Determination of liver tissue antioxidant enzyme activities

#### Estimation of superoxide dismutase (SOD) activity

Superoxide dismutase was calculated using the Kuthan, Haussmann [47] method. The capacity of the enzyme to block the phenazine methosulphate-mediated reduction of nitroblue tetrazolium dye is used in this experiment.

#### Estimation of glutathione peroxides (GPx) activity

Glutathione peroxides were calculated using the technique provided by Paglia and Valentine [48]. A solution comprising glutathione, glutathione reductase, and NADPH is mixed with liver tissue homogenate. The enzyme process is started by introducing the substrate, hydrogen peroxide, and measuring the A340. The rate of reduction in the A340 is directly related to the sample’s GPx activity.

### Estimation of liver tissue inflammatory markers

#### Determination of tumor necrosis factor alpha level

Tumor necrosis factor α levels in rat liver tissue were measured using rat TNF-enzyme-linked immunosorbent assay (ELISA) kits and the manufacturer’s instructions. TNF- monoclonal antibodies were precoated onto 96-well plates. Following the addition of samples and standards to the wells, biotinylated antibodies were added to each well. The Avidin-Biotin-Peroxidase Complex (ABC) was employed, together with a substrate solution, to create a blue hue that turned yellow with the addition of an acidic stop solution. Using a UV/visible ELISA plate reader, the optical density of the colored complex of the reaction mixture was recorded [49].

#### Determination of interleukin 1 beta level

The amount of IL-1β in rat liver tissue was evaluated using rat IL-1 ELISA kits and monoclonal anti-rat antibodies for IL-1β, as directed by the manufacturer. The absorbance is related to the quantity of rat IL-1 beta collected on the plate [50].

### Histopathological evaluation

Liver tissue samples were fixed in 10% neutral buffered formalin. Then dehydration with progressively higher grades of alcohol, clarifying with xylene, and embedding in paraffin. Tissue sectioning at 5 microns thick and staining with hematoxylin and eosin (H&E) [51].

### Immunohistochemistry of nuclear factor-kappa B in the liver tissue

Different animal groupings of liver tissues were cut into four-millimeter-thick parts. Deparaffinized sections were rehydrated, and endogenous peroxidase activity was inhibited using H2O2 in methanol. In a microwave, sections were pre-treated in citrate buffer (pH 6.0). At room temperature, sections were treated with monoclonal anti- NF-κB (Thermofisher Scientific, USA). The UltraVision Detection System (Thermo Scientific) was used to detect streptavidin peroxidase, followed by DAB and chromogen. Haematoxylin was used to counterstain the slides. The slides were examined under a light microscope to determine the amount of cell immunopositivity. The number of immunopositive cells in each slide was counted in five distinct microscopic fields, and the mean number for each slide was computed, followed by the mean SE for each group [52].

### Statistical analysis

The Shapiro-Wilks normality test revealed that all variables had a normal data distribution; hence, the parametric statistical analysis was carried out. Tukey’s multiple comparisons test was used to compare all variables using one-way ANOVA (GraphPad Prism, version 8.0.2). Statistical significance was judged to have been achieved when P was less than 0.05.

## Results

### Elevated plus maze test

The raised plus maze test showed a significant (*p* < 0.0001) decrease in the number of entries and time spent in open arms in rats induced by CCL_4_-induced liver failure. However, the number of entries and time spent in closed arms increased. The combination of tadalafil and *L. sativum* significantly increased time spent in open arms and closed arms compared to the CCL_4_-induced liver failure group. The number of rat entrances into closed arms was significantly reduced after treatment with the combination, unlike with separate medication treatments. However, time spent in closed arms increased significantly in CCL_4_-induced liver failure, tadalafil-treated, and *L. sativum*-treated groups, but there was no significant change when both tadalafil and *L. sativum* combinations were used compared to control rats (Table 1).

**Table 1:** Elevated plus maze behavioral assay

Treatment	Control	CCL_4_ liver failure	Tadalafil	Lepidium sativum	Combination (Tadalafil and LS)	*P* value
Number of enters in open arms	5 ± 0.79a	1.9 ± 0.33b	2.7 ± 0.3b	1.6 ± 0.18b	5 ± 0.5a	< 0.0001
Time spent in open rms (min)	82.90 ± 11.45a	20.50 ± 2.236b	50 ± 3.5c	15.10 ± 0.5099b	55 ± 3.5c	< 0.0001
Number of enters in closed arms	5.3 ± 0.7a	2 ± 0.3bc	2.9 ± 0.3c	1.6 ± 0.18c	4.6 ± 0.4ab	< 0.0001
Time spent in closed arm (min)	213 ± 18.07a	280 ± 3.5b	281 ± 3.6b	284.3 ± 2.4b	248.3 ± 1.8ab	< 0.0001

Means ± SE with different superscripts in the same row differ significantly (*P* < 0.05). CCL_4_ (Carbon tetrachloride) and LS (Lepidium sativum)

### **The effects of tadalafil and*L. sativum*alone or in combination on the activity of liver enzymes**

**Fig. 1:**
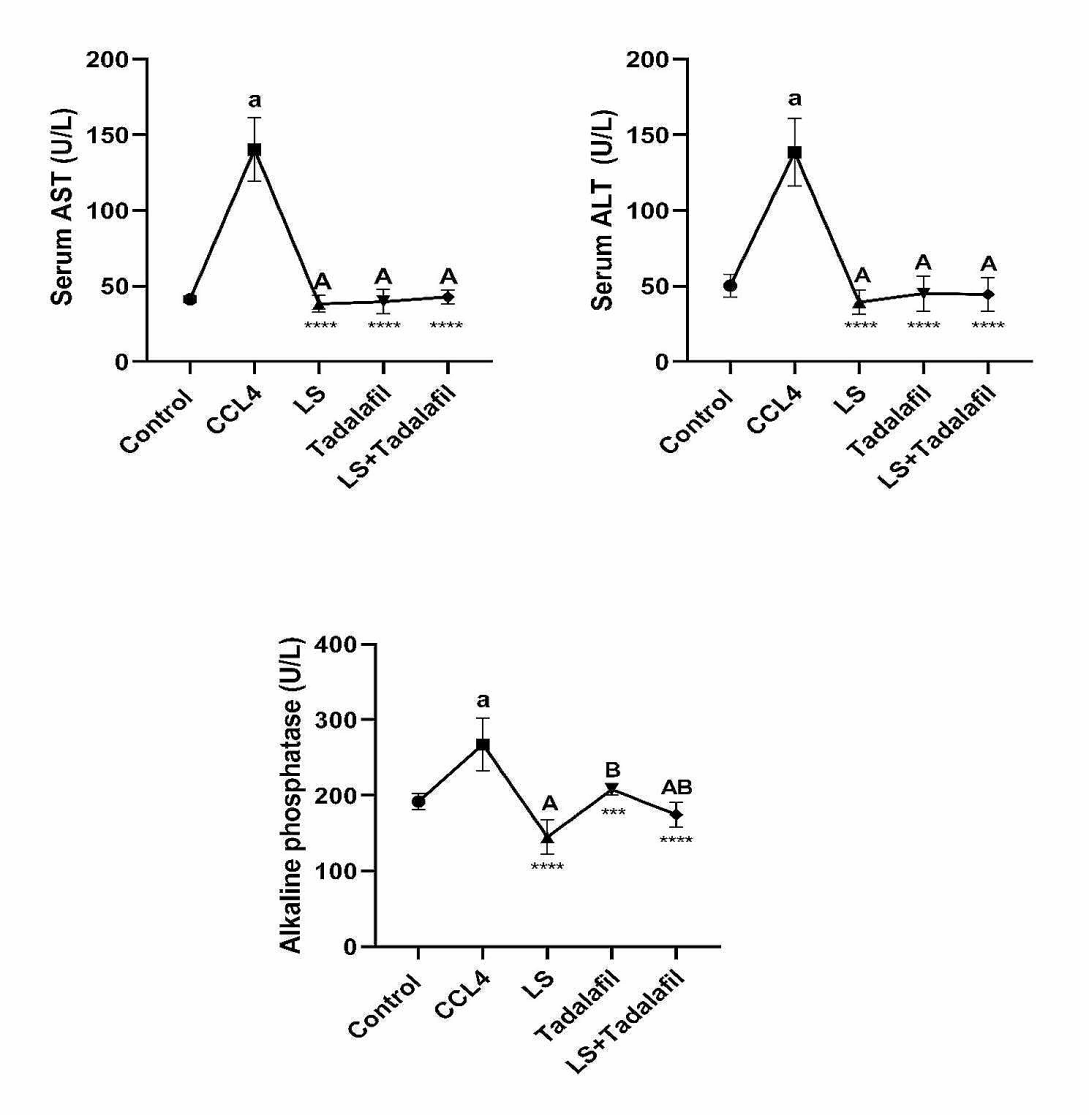
Effects of tadalafil and *L. sativum* (LS) alone or in combination on the activity of serum aspartate aminotransferase (AST), alanine aminotransferase (ALT) and serum alkaline phosphates (ALP) in carbon tetrachloride (CCL_4_) induced liver failure in rats. Note: ****p < 0.0001and ***p < 0.001 as compared to the CCL_4_ group. a *P* < 0.0001 as compared with the control group. Different large superscripts differ significantly (*P* < 0.05)

As indicated in Fig. 1, rats with CCL_4_-induced liver failure had higher levels of blood liver enzymes (AST, ALT, and ALP) than those with saline. Treatment with tadalafil *and L. sativum* led to a significant (*p* < 0.0001) drop in these enzyme levels. However, in acute liver failure induced rats, *L. sativum* -treated rats had lower ALP levels than tadalafil-treated groups. No significant difference was found in ALP serum levels between the combination and individual treatment groups.

### **The effects of tadalafil and*L. sativum*on liver proteins biomarkers, either alone or in combination**

**Fig. 2:**
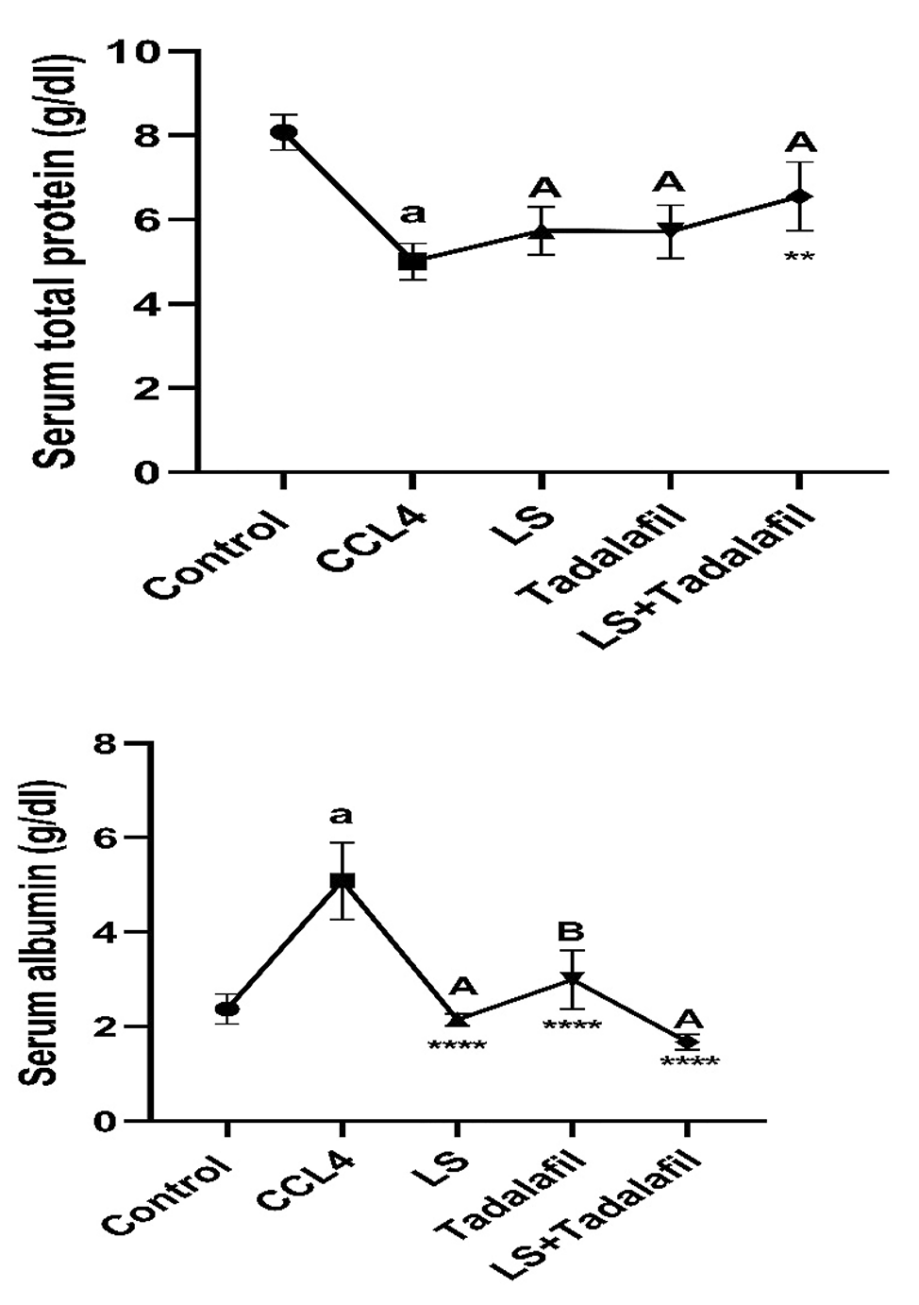
Effects of tadalafil and *L. sativum* (LS) alone or in combination on the serum albumin and total protein levels in carbon tetrachloride (CCL_4_) induced liver failure in rats. Note: ****p < 0.0001and **p < 0.01 as compared to the CCL_4_ group. a *P* < 0.0001 as compared with the control group. Different large superscripts differ significantly (*P* < 0.05)

As shown in Fig. 2, the CCL_4_-induced liver failure group had considerably greater (*p* < 0.0001) serum albumin levels than the control saline-treated group, whereas total protein serum levels were significantly lower. There was a significant (*p* < 0.0001) decline in serum albumin levels after treatment with tadalafil and *L. sativum* alone or in combination with CCL_4_-induced liver failure, with a greater drop in the *L. sativum-*treated group and combination-treated group. Furthermore, in CCL_4_-induced liver failure, total protein serum levels increased significantly (*p* < 0.001) only following treatment with the tadalafil- L. sativum combination.

### **The effects of tadalafil and*L. sativum***, **either alone or in combination on serum bilirubin (direct, total) and gamma-glutamyl transferase levels**

**Fig. 3:**
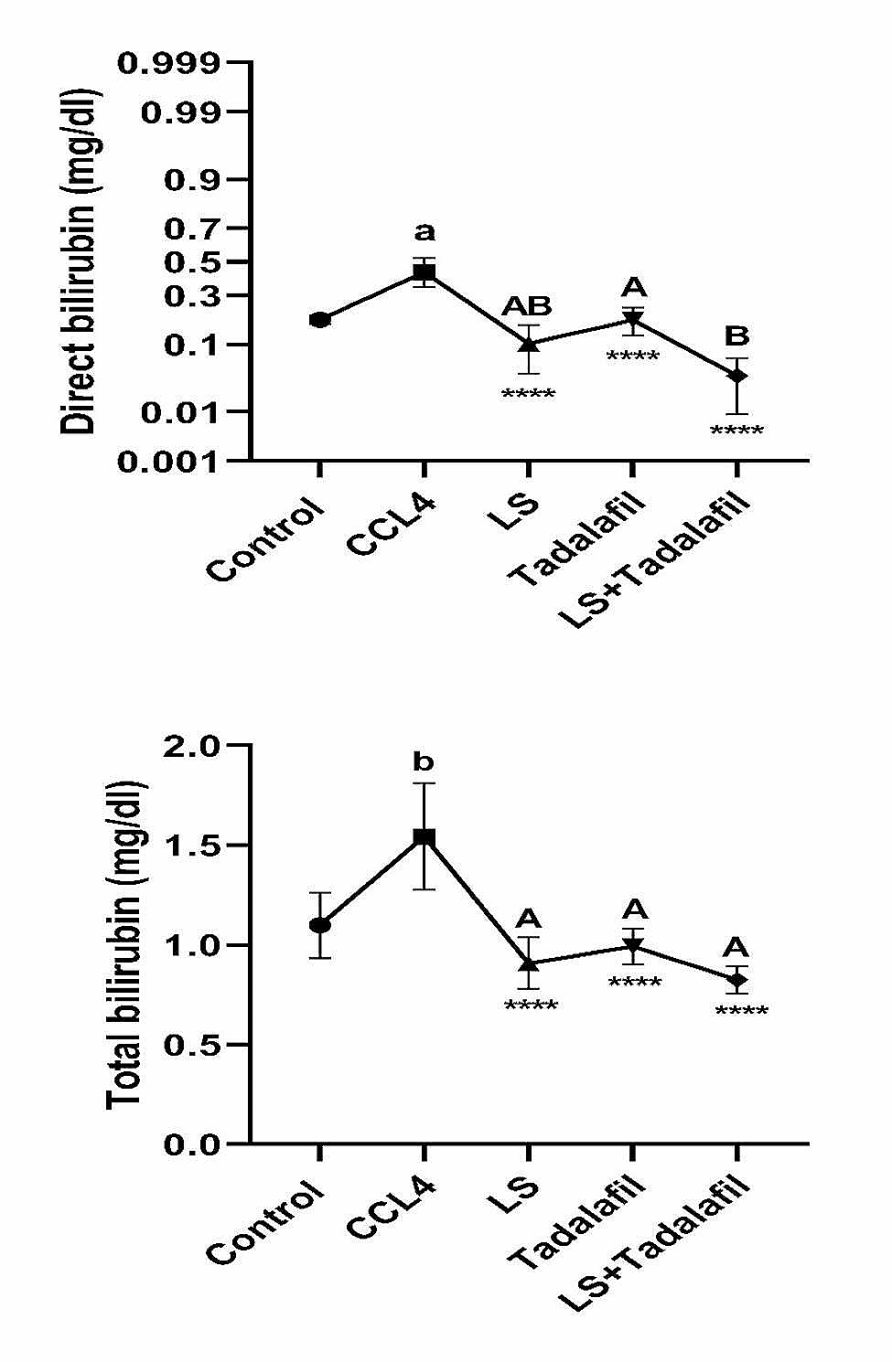
Effects of tadalafil and *L. sativum* (LS) alone or in combination on the serum bilirubin levels (direct and total) in carbon tetrachloride (CCL_4_) induced liver failure in rats. Note: ****p < 0.0001 as compared to the CCL_4_ group. a *P* < 0.0001 as compared with the control group. Different large superscripts differ significantly (*P* < 0.05)

Figure 3 shows that the CCL_4_-induced liver failure group had considerably higher (*p* < 0.0001) levels of blood-direct bilirubin and significantly higher (*p* < 0.001) levels of serum total bilirubin and GGT than the control saline-treated group. While all treated groups in CCL_4_-induced acute liver failure showed a significant (*p* < 0.0001) decrease in serum levels of the previously mentioned parameters when compared to the CCL_4_-induced liver failure, there was no significant difference in lowering serum total bilirubin and GGT serum levels across treated groups, with the exception of the tadalafil-*L. sativum* combination-treated group, which showed a significant (*p* < 0.05) decrease in serum direct bilirubin levels.

### **The effects of tadalafil and*L. sativum***, **either alone or in combination on oxidative stress and antioxidant parameters**

**Fig. 4:**
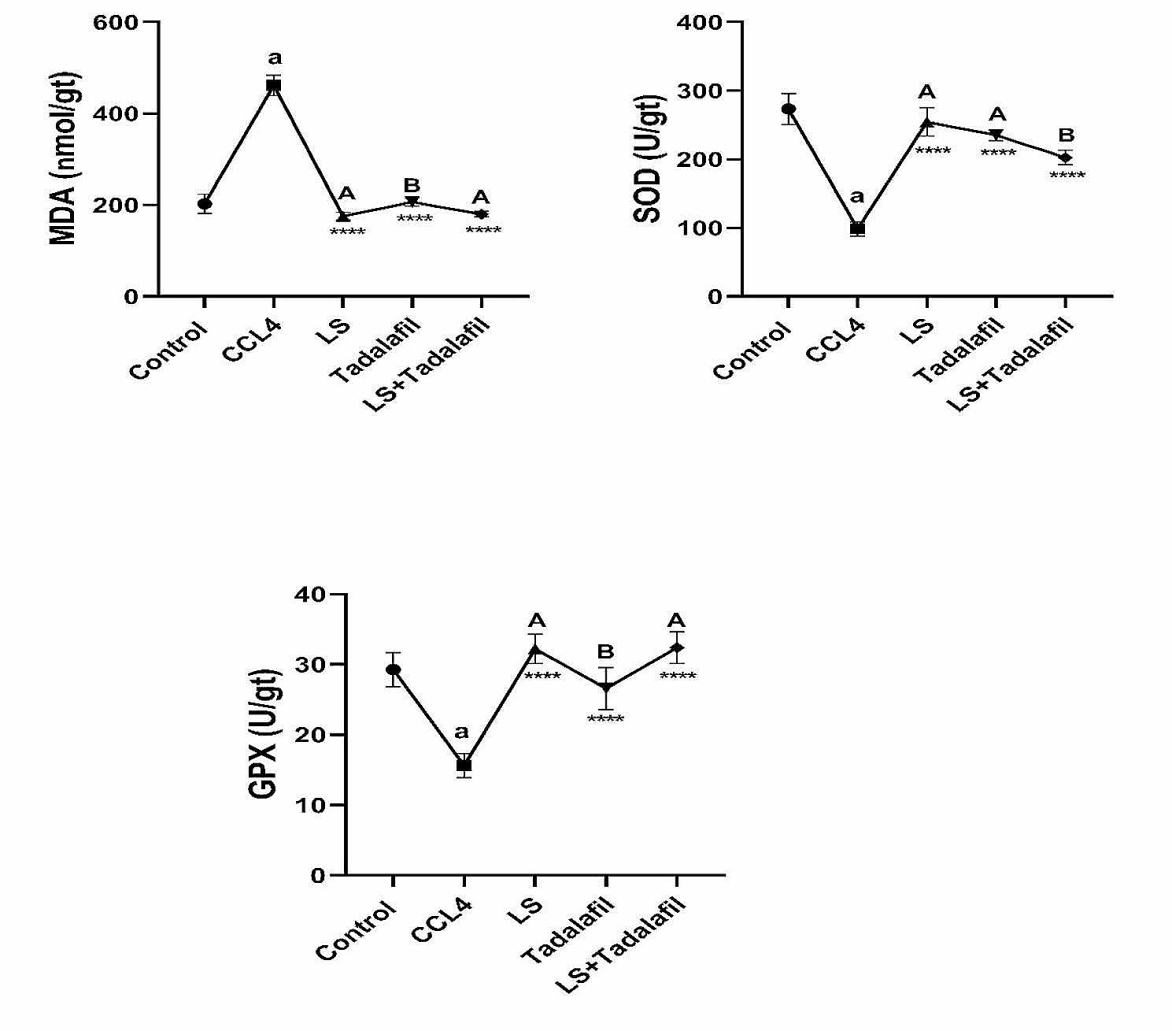
Effects of tadalafil and *L. sativum* (LS) alone or in combination on the tissue malondialdehyde (MDA), superoxide dismutase (SOD) and glutathione peroxidase (GPx) activity in carbon tetrachloride (CCL_4_) induced liver failure in rats. Note: ****p < 0.0001 as compared to the CCL_4_ group. a *P* < 0.0001 as compared with the control group. Different large superscripts differ significantly (*P* < 0.05)

Figure 4 reveals that the CCL_4_-induced liver failure group had considerably greater levels of MDA in the liver tissue (*p* < 0.0001) and significantly lower levels of tissue SOD and GPx activity (*p* < 0.0001) than the control saline-treated group. When compared to CCL_4_-induced liver failure, all treated groups demonstrated a substantial (*p* < 0.0001) drop in tissue levels of MDA and a significant (*p* < 0.0001) increase in tissue levels of SOD and GPx. Interestingly, the *L. sativum*-treated group and the tadalafil-*L. sativum* combination-treated group improved the most in MDA and GPx activity in the tissues.

### **The effects of tadalafil and*L. sativum***, **either alone or in combination on cellular inflammatory mediators**

**Fig. 5:**
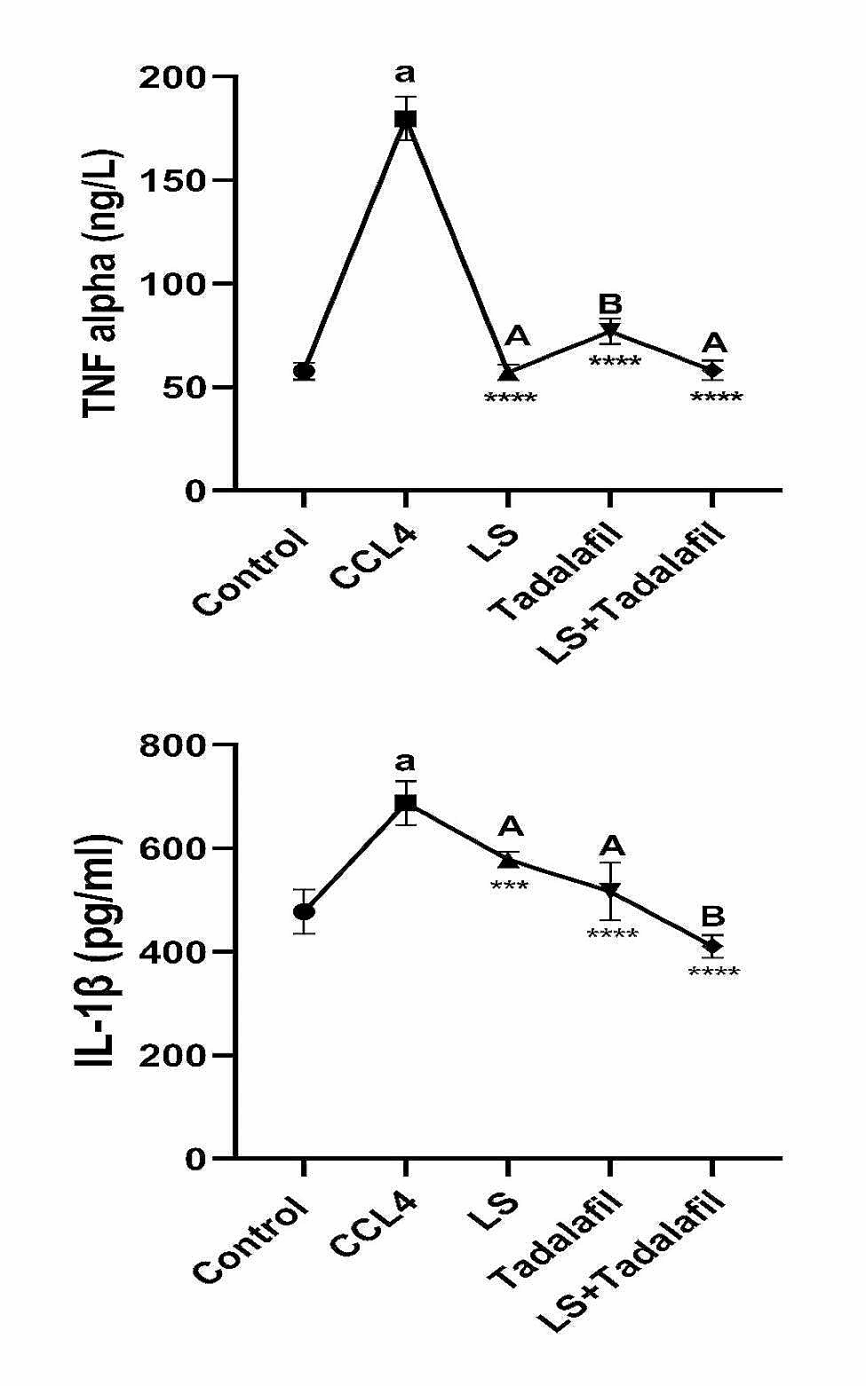
Effects of tadalafil and *L. sativum* (LS) alone or in combination on the tissue interleukin 1 beta (IL-1β) and tumor necrosis factor- alpha (TNF-α) in carbon tetrachloride (CCL_4_) induced liver failure in rats. Note: ****p < 0.0001 and ***p < 0.001 as compared to the CCL_4_ group. a *P* < 0.0001 as compared with the control group. Different large superscripts differ significantly (*P* < 0.05)

Figure 5 shows that the CCL_4_-induced liver failure group had significantly higher levels of IL-1β and TNF-α in the liver tissue (*p* < 0.0001) than the control saline-treated group. Treatment with *L. sativum* and tadalafil, either alone or in combination, resulted in a significant (*p* < 0.0001) decrease in IL-1β and TNF-α levels in the liver tissue when compared to the CCL_4_-induced liver failure group, with a possibly superior effect for the tadalafil-*L. sativum* combination group.

### Histopathological evaluation

**Fig. 6:**
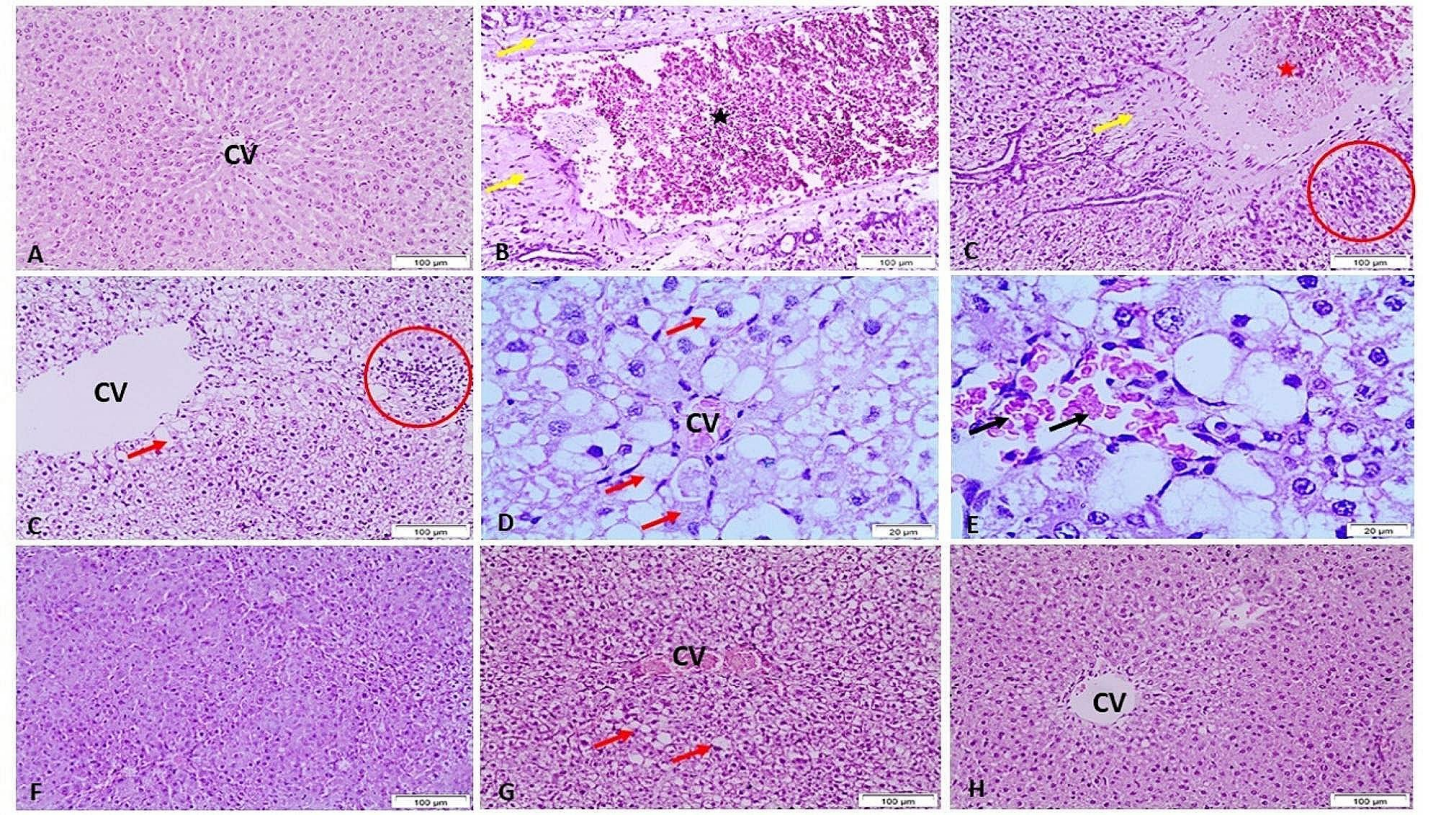
Representative micrograph of the liver of the studied group stained by HE. A) Control negative group showing the normal architecture of the liver. B-E) CCL_4_-treated group showing congestion of the central vein (CV), congestion of the portal blood vessels (black star), thrombosis of the portal blood vessels (red star), perivascular fibrosis (yellow arrows), focal areas of mononuclear cell infiltration (red circles), vacuolar degeneration of hepatocytes (red arrows) and hemorrhage (black arrows). F) CCL_4_ + *L. sativum*-treated group showing improvement of the liver cells with some vacuolar degeneration. G) CCL_4_ + tadalafil showing vacuolar degeneration (red arrows) and congestion of central veins (CV). H) CCL_4_ + *L. sativum* + tadalafil-treated group showing improvement of the liver cells with normal architecture

The lesion score of the testicular histopathology data in all examined groups was summarized in Table 2. The liver architecture in the control negative group was found to be normal. CCL_4_ treatment resulted in either vascular or parenchymal alterations. The vascular alterations include blood vessel thrombosis and perivascular congestion. Hepatocyte vacuolar degeneration and localized regions of mononuclear cells were the hepatocellular alterations. The liver tissues improved in the CCL_4_ and *L. sativum*-treated group; however, there was considerable vacuolar degradation of the hepatocytes. In some cases, CCL_4_ + tadalafil indicated vacuolar degeneration of hepatocytes as well as congestion of the central veins. The CCL_4_ + *L. sativum* + tadalafil-treated group improved liver cells with the normal architecture (Fig. 6).

**Table 2:** summarized the lesion score of the studied groups

Groups Lesions	Control -ve	CCL_4_	CCL_4_ + LS	CCL_4_ + T	CCL_4_ + LS + T
Congestion of CV	-	+++	+	+	+
Congestion of portal bl.vs	-	++	+	+	-
Thrombosis of portal bl.vs	-	+	-	-	-
Perivascular fibrosis	-	++	-	-	-
Vacuolar degeneration	-	+++	+	++	-
Mononuclear cell infiltration	-	++	-	-	-

- No lesions, + lesions present in 2–3 sections, ++ lesions present in 4–7 sections, +++ lesions present in 8–10 sections

-CCL_4_ (carbon tetrachloride), LS (Lepidium sativum), T (tadalafil)

### Immunohistochemistry analysis

**Fig. 7:**
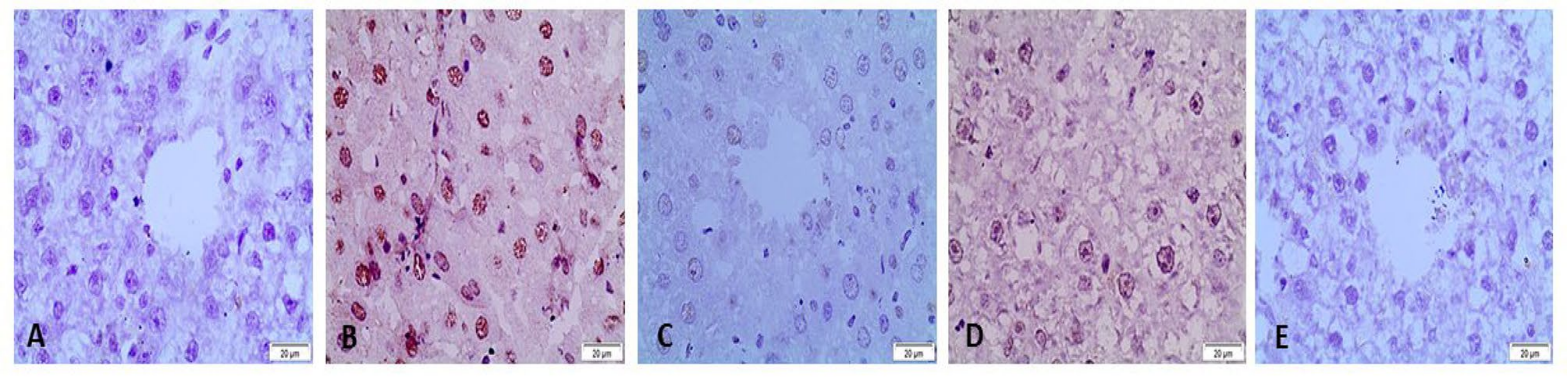
Immunohistochemical staining of nuclear factor-Kappa B (NF-κB) in rat liver. A) Control negative group showing no expression of NF-κB. B). CCL_4_-treated showed significant increase in NF-kB immunoreactivity in the cytoplasm of hepatocytes. The brown color indicates NF-kB positivity. C) CCL_4_ + *L. sativum*-treated-group showing significant reduction in NF-kB immunostaining. D) CCL4 + tadalafil-treated group showing significant reduction in NF-κB immunostaining. E) CCL_4_ + *L. sativum* + tadalafil treated-group showing no expression of NF-κB.

Nuclear factor-kB immunohistochemical staining in rat liver. CCL_4_ treatment significantly increased NF-κB immunoreactivity as compared to CCL_4_ + *L. sativum* and CCL_4_ + tadalafil-treated groups. There was no expression in the CCL_4_ + *L. sativum* + tadalafil and control negative groups (Fig. 7; Table 3).

**Table 3:** Effects of Lepidium sativum, tadalafil and mixture of both treatment on the percentage expression of nuclear factor-kB (NF-κB), in the liver of rats exposed to CCL_4_ hepatotoxicity

Groups	Control -ve	CCL_4_	CCL_4_ + LS	CCL_4_ + T	CCL_4_ + LS + T
NF-κB	N	19.75 ± 0.13 ^a^	3.12 ± 0.3 ^b^	4.85 ± 0.1 ^b^	N

^a, b^ values are not sharing a common superscript letter differ significantly at *P* < 0.05 .CCL4 (carbon tetrachloride), LS (Lepidium sativum), T (tadalafil)

## Discussion

Liver failure can occur as a consequence of viral contamination, too much drug use, alcohol addiction, and exposure to numerous harmful substances [53]. The hepatotoxic experimental rat model of CCL_4_ damage is physiologically and pathologically remarkably comparable to human hepatotoxic liver injury [54]. CCL_4_ damages the liver by causing oxidative cellular stress, peroxidation of lipid membranes, and inflammation [55]. The cytochrome P2E1 enzyme in the liver converts CCL_4_ to the hazardous reactive trichloromethyl and trichloromethyl peroxide radicals [56]. These reactive radicals subsequently attach to unsaturated fatty acids in the membranes of hepatocytes, mitochondria, and the endoplasmic reticulum, initiating a chain lipid peroxidation process that causes hepatocyte and intracellular structural damage and death [57].

Carbon tetrachloride is a potent hepatotoxin that can cause organ problems like liver fatty layer degeneration and centrilobular necrosis [58]. Its hepatotoxic effect is characterized by increased liver-damaging enzymes and pathological abnormalities. Normalization of these enzymatic parameters indicates improved liver function, while hepatotoxicity is measured by alterations in transaminase and phosphatase levels [59, 60]. High levels of AST indicate compromised liver function, similar to viral hepatitis, cardiac infarction, and muscular damage. ALT enzymes convert alanine to pyruvate and glutamate, released from hepatocytes into the blood in liver diseases. Elevated ALP indicates liver blockage or illness [34]. Serum bilirubin levels are elevated due to leakage from hepatocytes to plasma, which is generally caused by hepatic obstruction to bile outflow and cholestasis [61].

The current study found that both tadalafil and *L. sativum* had anxiogenic effects when administered alone in CCL_4_-induced liver failure in rats; however, the combination-treated group appeared to have anxiolytic effects by increasing the length and number of entries in open arms. Furthermore, the number of entries reduced in closed arms for both tadalafil and *L. sativum* when given alone in CCL_4_-induced liver failure in rats and reverted to the normal levels in combination-treated rats with decreased time spent.

The elevated plus maze is a classic rat behavioral test that has been validated for studying the anti-anxiety effects of pharmacological medications [62]. Anxiolytic drugs preferentially enhance exploration of the open arms while decreasing exploration of the enclosed arms, and anxiogenic drugs selectively reduce exploration of the open arms while increasing exploration of the closed arms. Also, in the elevated plus maze anxiety test, there is a growing notion that NO may cause anxiety [63]. The NO-cGMP pathway is well known to regulate anxiety in rats. However, research is mixed as to whether stimulating the NO-cGMP pathway increases or decreases anxiety-like behavior.

The bulk of research indicates that inhibiting the NO-cGMP pathway is anxiolytic and activating it is anxiogenic [64]. Chronic sildenafil or tadalafil administration has antidepressant-like effects in rats, but only when combined with muscarinic receptor antagonism [65]. According to Balgoon [66], *L. sativum* therapy in Alzheimer’s disease-induced rats reduced elapsed time significantly, indicating better memory and learning. The memory and learning benefits of LS shown in this study might be attributed to lower acetylcholinesterase activity, which improves cholinergic neurotransmission.

The current study found that *L. sativum* and tadalafil, particularly the combination group, normalized high levels of AST, ALT, ALP, and bilirubin and decreased total protein activity in an orogastric CCL_4_-induced liver failure rat model. Similarly, according to Al-Asmari, Athar [34] research, pretreatment with *L. sativum* seeds and silymarin reduces liver damage induced by intraperitoneally injected CCL_4_ by lowering AST, ALT, ALP, and bilirubin levels. In addition, Rajab and Ali [35] study found that a 12-week pretreatment with *L. sativum* extract effectively reduced liver damage induced by intraperitoneal CCL_4_ injection by reducing blood liver enzymes and inflammatory biomarkers. Both of the previous investigations looked at the preventive impact of *L. sativum* against CCL_4_-induced liver damage, whereas the current study looked at the potential therapeutic effectiveness.

Tadalafil’s activation of cGMP-dependent protein kinases causes vasodilation, anti-inflammatory, and anti-proliferative effects, as well as a decrease in collagen formation [18, 67]. PDE5 inhibitors have been found to have anti-fibrotic properties as well as a potentially promising function in the treatment of inflammatory diseases [25]. Similarly to our present investigation, Broermann, Schmid [25] discovered that anti-fibrotic effects caused by the PDE5 inhibitor are represented by differently expressed miRNAs in the liver and reduce CCL_4_-induced chronic liver failure in rats. Furthermore, fibrosis generated by thioacetamide injection twice weekly for 6 weeks in rats was mitigated by tadalafil pretreatment via stabilization of inflammatory and fibrotic biomarkers [22]. However, in the current investigation, we demonstrated the therapeutic benefit of tadalafil in rats with acute liver failure caused by CCL_4_, particularly when combined with *L. sativum* extract, which showed a more significant therapeutic effect.

The oxidative stress caused by CCL_4_ deactivates cellular anti-oxidative enzymes, including peroxidase, catalase, and superoxide dismutase, which neutralize free radicals [60]. This leads to a buildup of O^2−^ and H_2_O_2_, causing liver damage. Lipid membranes are exposed to oxidative stress due to high levels of polyunsaturated fatty acids and transition metallic elements, which can damage cellular proteins, DNA, and inhibit antioxidant enzymes and degrade lipid membranes via the oxidative Haber-Weiss reaction [68].

The study found that CCL4 poisoning led to a significant increase in MDA levels and decreased antioxidant enzymes SOD and GPx activity. These alterations returned to normal after treatment with *L. sativum* and tadalafil, especially in combination. These findings are consistent with the findings of [22, 35]. Up-and-down inflammatory disorders accompany liver dysfunction/failure. TNF-α, a pro-inflammatory mediator, contributes to oxidative stress-induced liver damage, leading to apoptotic cell death and fibrosis. Kupffer cells release cytokines, chemokines, and pro-inflammatory mediators, initiating hepatic inflammation [60, 69]. In this study, CCL_4_ poisoning increased TNF-α and IL-1β levels. Treatment with *L. sativum* and tadalafil, in combination, reverses these alterations, demonstrating potent anti-inflammatory activity.

According to Toriumi, Horikoshi [70] research, diacylglycerol-O-(OH) is produced during the CCL_4_-induced liver damage process, culminating in activation of the protein kinase C/ NF-κB pathway and TNF-α mediated exacerbation of liver injury. Immunohistochemistry staining for NF-kB was significantly greater in the CCL_4_ treatment group compared to the CCL_4_ and *L. sativum* and CCL_4_ + tadalafil treatment groups in the current study. The CCL_4_ + *L. sativum* + tadalafil-treated group and control groups did not exhibit any expression. The NF-κB pathway is involved in the regulation of inflammatory responses as well as the control of apoptosis [71, 72].

Hepatotoxicity induced by CCL_4_ resulted in vascular and hepatic modifications such as thrombosis, congestion, vacuolar degeneration, and mononuclear cell infiltration in the current investigation. CCl_4_-induced liver damage is commonly used to evaluate hepatoprotective medicines. Hepatotoxicity is caused by the biotransformation of CCL_4_ into free radicals [73]. The liver, according to Veidal, Karsdal [74], is a target organ for CCL_4_ toxicity due to its function in the body’s defense through detoxification. Because CCL_4_ is a well-known hepatotoxic commercial solvent, it is used in a number of experimental models. In contrast to these minor hepatic modifications, subcutaneous injection of CCL_4_ (2 ml/kg b.w.) induced obvious hepatic necrosis, inflammation, fatty change, and fibrosis in rats after 12 weeks [75]. Furthermore, Hassanein, Al-Emam [76] noted that there were no major histological abnormalities in our investigation, including centrilobular necrosis and visible fatty alterations, due to the oral route and short period of CCL_4_ administration. With vacuolar disintegration of the hepatocytes, the liver tissues improved in the CCL_4_ and *L. sativum*-treated groups. CCL_4_ and tadalafil caused vacuolar degeneration of hepatocytes as well as central vein congestion. CCL_4_, *L. sativum*, and tadalafil treatment groups enhanced the appearance of liver cells. These findings are consistent with those of [22, 34].

## Conclusion

Tadalafil and *L. sativum* pre-treatment of CCL_4_-exposed rats effectively improved liver function, restored the liver’s redox stability and cyto-functionality by decreasing lipid peroxidation, boosting anti-oxidants such as GPx and lowering inflammatory cell mediators such as IL-1β and TNF-α. This study is essential in directing the scientific community toward identifying the positive effect of natural phytochemicals on lowering the health concerns associated with CCL_4_, a prevalent environmental contaminant. Moreover, the findings pave the way for the future use of plants in combination with new pharmaceuticals in the treatment of acute liver failure. Furthermore, additional investigations in different animal models of liver failure are required to provide insight into the specific molecular mechanisms driving *L. sativum* and tadalafil’s therapeutic effectiveness in liver failure.

## Data Availability

The datasets generated during and/or analyzed during the current study are available from the corresponding author on reasonable request.
